# An evolutionary mismatch narrative to improve lifestyle medicine: a patient education hypothesis

**DOI:** 10.1093/emph/eoab010

**Published:** 2021-02-24

**Authors:** Anthony J Basile, Michael W Renner, Brandon H Hidaka, Karen L Sweazea

**Affiliations:** 1 School of Life Sciences, Arizona State University, 427 E Tyler Mall, Tempe, AZ 85287, USA; 2 Center for Evolution and Medicine, Arizona State University, 427 E Tyler Mall, Tempe, AZ 85287, USA; 3 Department of Family Medicine, Mayo Clinic Health System, 1400 Bellinger St., Eau Claire, WI 54703, USA; 4 College of Health Solutions, Arizona State University, 550 N 3rd St, Phoenix, AZ 85004, USA

**Keywords:** evolutionary mismatch, patient education, lifestyle medicine, behavior change

## Abstract

An evolutionary perspective provides a unifying explanation for the modifiable risk factors and lifestyle-based interventions for the leading causes of morbidity and mortality globally. Non-communicable diseases develop from an evolutionary mismatch between the prior environment and modern patterns of behavior; however, it is unclear whether an evolutionary mismatch narrative could promote positive behavior change in patients. We hypothesize that educating patients about evolutionary mismatch could augment efforts to improve healthful behavior. Specifically, explaining the ‘why’ behind what is being recommended could promote health literacy and adherence. Furthermore, we offer suggestions of how clinicians could educate patients about evolutionary mismatch for key-lifestyle factors, diet and physical activity, as well as several specific modern diseases. We also consider how to sidestep patients’ skepticism of evolutionary theory. Here, we lay the groundwork for research on how educating patients with an evolutionary mismatch narrative could impact health behaviors and improve outcomes.

## INTRODUCTION AND CLINICAL NEED

Clinician recommendations do not always translate directly into patient behavior change [1]; however, patient education remains a necessary step for behavior change [2]. It is possible that an evolutionary medicine (EM) perspective may promote positive behavior change. EM applies evolutionary theory to better understand why humans are vulnerable to diseases and how to improve the prevention and treatment of those diseases [3]. For example, EM principles have shown great promise in transforming cancer treatment [4] and management of antibiotic resistance [5]. However, there is a glaring gap between the use of EM in research settings and the implementation of EM principles into lifestyle medicine and behavior change.

Clinicians can use EM principles in their patient education to increase health literacy. The Institute of Medicine defines ‘Health Literacy’ as the degree to which individuals have the capacity to obtain, process, and understand basic health information and services to make appropriate health decisions [6]. Two types of evidence increase health literacy among patients: statistical and narrative evidence. Statistical evidence provides quantitative information, whereas narrative evidence presents a cohesive story to educate the patient [7]. A meta-analysis comparing the effectiveness of these two educational strategies revealed that narrative evidence produces greater intentions for behavioral change among patients [8]. Moreover, strong evidence shows narrative interventions actually increase health-promoting behaviors [9]. We propose that adding an evolutionary perspective to narrative evidence could augment behavior change, which has not been directly tested.

Elucidating the ultimate causes of disease may empower patients to apply EM to their lifestyle in areas where mechanistic explanations have fallen short in the past. Evolutionary mismatch provides a unifying framework for understanding the epidemiological patterns and lifestyle-based treatments of the most prevalent ‘diseases of civilization’ (e.g. type 2 diabetes, cardiovascular disease, obesity; [10]). Here, we explore the hypothesis that providing an evolutionary narrative behind pathologies arising from evolutionary mismatch will grant patients a deeper understanding that will in turn facilitate meaningful behavior change. We describe how education on evolutionary mismatch might motivate behavior change by increasing adherence to various lifestyle prescriptions. We then provide practical materials for clinicians to incorporate evolutionary theory into their practice while avoiding controversy by tailoring explanations to a patient’s particular level of evolutionary acceptance. Finally, we consider how future investigators might implement this behavior change hypothesis into a research setting.

## SUPPORT FOR EVOLUTIONARY MISMATCH EDUCATION

The field of medicine has largely focused on the proximate, or mechanistic, causes of many chronic diseases. However, an evolutionary perspective explores why we are vulnerable to disease in the first place. For example, the main mechanistic cause of obesity is a surplus of calories, whereas numerous evolutionary hypotheses have been presented for its ultimate cause, such as humans’ ability to store calories for potential future famines [11]. While the effects of evolutionary mismatch education on medical patients has yet to be tested, several EM advocates have stated the potential benefits of educating patients on EM or mismatch (see Table 1). Future research is needed to assess the efficacy of this education.

**Table 1.** Support in the literature for educating patients on evolutionary mismatch

Author	Excerpt
Enam and Hasmi, 2018 [46]	*‘It is…doctors (and their patients) to whom understanding the evolutionary basis of disease is most relevant’.*
Eaton and Eaton, 2017 [47]	*‘The public has been told ad nauseum that exercise and weight control are essential for preventing T2DM, but they have not been provided an understandable, convincing link between these factors and T2DM’s biological basis. An evolutionary perspective, together with the concept of insulin receptor competition, may fill that need’.*
Perlman, 2013 [3]	*‘…evolutionary explanations of disease are important because patients often want to know why they have the diseases they have’.*
Perlman, 2011 [48]	*‘Nonetheless, understanding that manifestations of disease may be adaptations provides a richer understanding of these manifestations, and it may be helpful to patients to learn that their symptoms, though distressing, are part of their healthy coping with their disease’.*
Naugler, 2009 [49]	*‘…a basic knowledge of evolutionary medicine might help in explaining the causation of diseases to patients’.*
Eaton *et al*., 2002 [50]	*‘…providing accurate health advice is less than half the battle; at least as important is achieving patient compliance. Providing an explanation for health promotion based on a coherent theory of how disease arises from the mismatch between our original design and our current circumstances should help’.*

The idea of evolutionary mismatch has not been confined to academia. Since the early 2000s, there has been growing interest in nutritional and exercise guidelines intended to mimic those of our ancestors, commonly known as the ‘Paleo’ or ‘Ancestral Health Movement’ [12]. The Modern Paleo Diet (MPD) is a food-group-based approach to health improvement which calls for the elimination of any foods that were not consumed by ancestral hunter-gatherers [13, 14]. Konner and Eaton [15] applied the mismatch framework to humans and developed the ‘evolutionary discordance hypothesis’, which states that the prevalence of chronic disease has increased due to a departure from the hunter-gatherer lifestyles for which we are well-adapted. The MPD is a direct application of the evolutionary discordance hypothesis [10, 16]. While there is controversy surrounding the diet’s evolutionary justification (including the fact that there is no singular ancestral diet) [17, 18], the popularity of this movement indirectly illustrates how a patients’ understanding of the mismatch hypothesis can promote changes in physical activity and nutritional behaviors [19]. Though the MPD is grounded in evolutionary theory, only four studies explicitly reported giving participants an evolutionary explanation (see Supplementary Table S1) [20–23]. MPD studies that did not specifically report giving participants an evolutionary rationale still showed physiological improvements [24–26]. Thus, the impact of mismatch education on behavior change is plausible, but unproven; nevertheless, the popularity of this dietary pattern highlights the public’s appetite for an evolutionary basis for health advice.

## LIFESTYLE BEHAVIOR CHANGE VIA AN EVOLUTIONARY MISMATCH NARRATIVE

Data on the ability of lifestyle education alone to affect meaningful behavior change is mixed. A 2005 meta-analysis examined the efficacy of lifestyle intervention education in adults with diabetes and found that the intervention group had a 50% lower risk of incidence of type 2 diabetes after a 1-year follow-up [27]. In contrast, a 2013 meta-analysis found no evidence for improvement in all-cause mortality or cardiovascular outcomes in adults with type 2 diabetes following an education-based lifestyle intervention [1]. Furthermore, in a systematic review of the impact of knowledge of genetic risk on behavior, Heshka *et al*. [28] report that current methods of patient education are insufficient to induce favorable outcomes and that improved education strategies are needed. An evolutionary narrative can serve as a novel education strategy for translating education into behavior change.

To our knowledge, there is a single study that explored how evolutionary mismatch education impacts intentions to change behavior. Sherry (2018) [29] found that a one-time brief introduction to evolutionary biology produced a shift in high school students’ perceptions of healthy eating and led to intended dietary changes. Although, actual behavior was not measured. Sherry (2018) [29] found that explaining nutrition in an evolutionary context was necessary to change students’ perceptions of healthy eating. Our hypothesis builds upon these encouraging results.

## STRATEGIES FOR CLINICIANS TO IMPROVE ADHERENCE TO BEHAVIOR CHANGE PRESCRIPTIONS

### The transtheoretical model (stages of behavior change)

**Figure 1.**
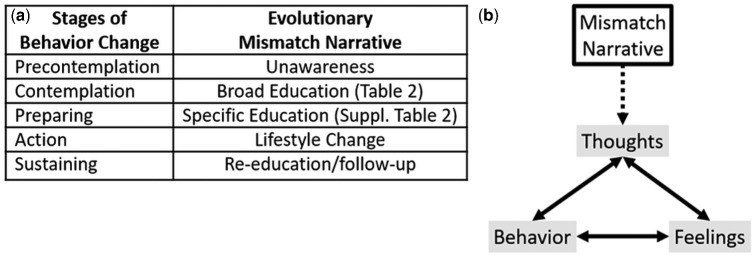
Applying evolutionary mismatch narrative to (**a**) the stages of behavior change and (**b**) the Cognitive Behavior Change Model (CBT).

There are many ways clinicians could apply evolutionary mismatch education in clinical settings. Pairing the evolutionary mismatch narrative with evidence-based behavior change models may be an effective strategy. The transtheoretical model (TTM) describes a sequence of cognitive and behavioral steps that individuals take to change behavior ([30]; see Fig. 1a). Much work has been done to apply the TTM to nutrition and diet change [31]. A recent 2017 study found that dietary knowledge significantly motivated participants to move into later stages of behavior change, which in turn improved the outcome of glycemic control among patients with type 2 diabetes [32]. As Sherry (2018) suggests, an evolutionary perspective may cognitively promote progression in stages of behavior change. An evolutionary narrative may impact several stages of behavior change: a patient’s attitude, perception of specific behaviors, and increase self-efficacy, all of which are the precursors to lifestyle modification [33].

### Cognitive behavior therapy model

**Figure 2.**
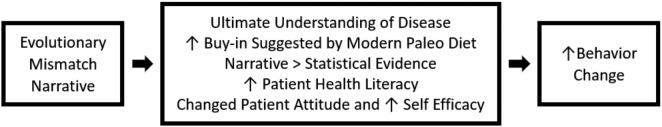
Summary of the evidence supporting the use of an evolutionary mismatch narrative to promote behavior change.

Evidence for Cognitive Behavior Therapy’s (CBT) effects on changing behavior is strong [34, 35]. Briefly, CBT is a psychotherapy treatment method that improves health behaviors by changing the way one thinks. Numerous studies have highlighted the effectiveness of CBT in improving diet, health [36, 37], and physical activity [38]. The ability of CBT to change behavior highlights the importance of education, which can alter a patient’s attitude and increase self-efficacy [33], an important step for behavior change. By explaining the ‘why’ of the patient’s disease, a mismatch narrative can help reframe a patient’s thoughts to influence their feelings and behavior in keeping with CBT (see Fig. 1b). In addition, due to the broadness of an evolutionary mismatch narrative, an integrative approach that incorporates other education techniques, specific to the patient, should be paired with this narrative to better promote behavior change [39]. A summary of the evidence to support the use of an evolutionary mismatch narrative can be found in Fig. 2.

## MISMATCH EDUCATION FOR DIET, PHYSICAL ACTIVITY AND VARIOUS DISEASES

Poor diet and low physical activity are strong risk factors for numerous diseases and addressing them can have a beneficial impact on disease risk or treatment. Often a ‘natural’ diet and lifestyle is thought to be healthful; however, what qualifies as ‘natural’ is unclear [40]. As Konner and Eaton (2010) [15] stated, the evolutionary (hunter-gatherer) perspective is the answer to what is a ‘natural’ diet and lifestyle for humans. Therefore, a diet and lifestyle that prevents mismatch disease would be a lifestyle that is natural. The intuitive nature of eating a ‘natural’ diet is easy to understand and lends itself to encouraging the consumption of whole, unprocessed foods that are widely recognized as inherently healthful [41]. Furthermore, sedentarism is a strong risk factor for metabolic disease that is also produced via mismatch [42]. Mismatch education may help to alleviate sedentarism by encouraging a physical activity regimen which resembles those prior to modern times. Moreover, this evolutionary mismatch perspective already aligns with numerous public health recommendations (e.g. promoting physical activity and consumption of minimally-processed whole foods; [43]). However, care must be taken to avoid the misapplication of an evolutionary perspective; for example, low-glycemic foods promoted by the MPD has been taken to an extreme and interpreted by some to mean an ultra-low carbohydrate (e.g. a carnivore diet). In rushed encounters between clinicians and patients, important nuances in dietary advice may be missed.

**Figure 3.**
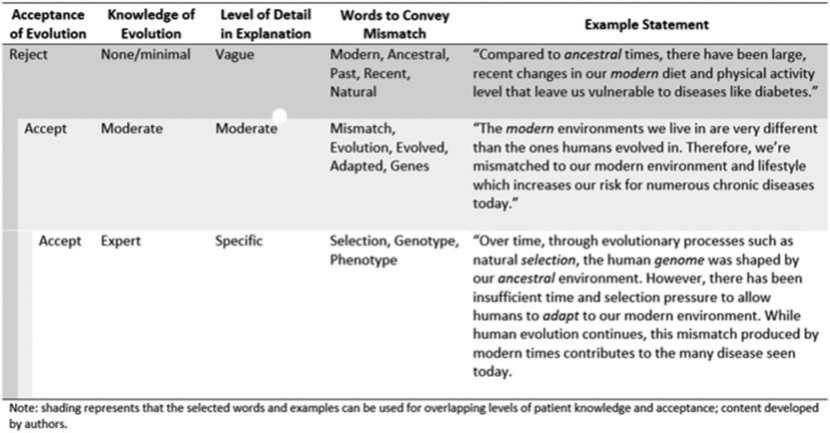
Examples of words and statements and level of detail within explanations of an evolutionary mismatch narrative based on a patient’s acceptance of evolution and level of knowledge.

The implications of a mismatch narrative extend well past just diet and physical activity as mismatch contributes to numerous diseases. Supplementary Table S2 presents explanations for mismatch diseases that clinicians may find useful in explaining the specific nature of a disease to each patient. Also included is supporting evidence that treatment based on the evolutionary mismatch hypothesis is effective in alleviating each condition. A clinician could use the broader mismatch as a hook (Fig. 3) and then focus on a specific disease (Supplementary Table S2). Moreover, specific phrases can be used within patient education that convey or imply mismatch without specifically talking about evolution. Pairing examples from Supplementary Table S2 with behavior change models (Fig. 1) can provide patients with a new understanding of their diagnosis and potentially motivate them to adhere to clinicians’ recommendations. It should be noted that clinicians often recognize that education alone does not necessarily lead to behavior change, but education, *per se*, can be effective for some patients. To determine the effectiveness of this evolutionary mismatch narrative education, future research should compare this narrative to other education interventions and measure behavior change.

## POTENTIAL LIMITATIONS DUE TO CONTROVERSY SURROUNDING EVOLUTION

Despite overwhelming evidence and scientific consensus, many US citizens remain unconvinced or opposed to the theory of evolution by natural selection [44]. This disconnect is an important barrier to incorporation of evolutionary explanations into patient education. For example, a survey of nutrition and dietetics professionals and students found that less than half reported that they were likely to provide an evolutionary explanation of a condition or disease to a patient or client [45]. We therefore explore how the mismatch narrative can be tailored to avoid provoking controversy among patients with barriers to acceptance of evolution. Fig. 3. outlines examples of words and statements for explaining the evolutionary narrative to patients with varying levels of evolutionary acceptance. Clinicians can still communicate evolutionary mismatch without invoking controversial perspectives that might impede patient adherence or jeopardize the patient-clinician relationship. For example, to convey change over time, a comparison between the ancestral and modern environment could be used without mentioning evolution (see Fig. 3). A clinician can solely use language intended for people who reject evolution to avoid any possible conflict. Evolutionary mismatch education does not require either the clinician or patient to have a comprehensive understanding of evolutionary theory. However, we do recognize that some clinicians may unintentionally apply evolutionary theory to excuse racist/eugenic ways of thinking. This underscores the need for including accurate evolutionary theory in medical education as well as recognition of the ableist and racist past of the medical field through misuse of evolutionary theory.

## CONCLUSIONS

Evolutionary mismatch education may improve patients’ health behavior by deepening their understanding. While not always sufficient, education is a necessary step of lifestyle change. Reframing the patient’s perspective by providing an evolutionary mismatch narrative to focus on the ultimate cause—rather than just a mechanistic explanation—of a chronic disease may motivate beneficial lifestyle behavior change because patients will understand the theory behind their lifestyle prescriptions. Although evolutionary theory can be polarizing, there are simple ways that clinicians can sidestep this controversy. Given the burden of chronic diseases of civilization, research exploring this hypothesis has broad implications.

## Supplementary data

Supplementary data is available at *EMPH* online.

## Supplementary Material

eoab010_Supplementary_Data
