# Supplementary material for: An evolutionary mismatch narrative to improve lifestyle medicine: a patient education hypothesis
Source: Evol Med Public Health. 2021 Feb 24;9(1):eoab010. doi: 10.1093/emph/eoab010 (PMC7962761; doi:10.1093/emph/eoab010)
Supplement: eoab010_Supplementary_Data [file eoab010_supplementary_data.zip › Supplemental Table 1_final.docx]

Supplemental Table 1. Examples of evolutionary mismatch education in clinical literature.

| **Author** | **Excerpt** | **Conclusion** |
| --- | --- | --- |
| Jönsson et al. 2013 [1] | *“The evolutionary rationale for a Paleolithic diet and potential benefits were explained.”* | A Paleolithic diet is more satiating per calorie than a diabetes diet in patients with type 2 diabetes. The Paleolithic diet was seen as instrumental in weight loss, albeit it was difficult to adhere to. |
| Jönsson et al. 2010 [2] | *“Only subjects in the Paleolithic group were educated in the concept of evolutionary health promotion and the potential benefits of a Paleolithic diet.”* | A Paleolithic diet is more satiating per calorie than a Mediterranean-like diet. |
| Jönsson et al. 2009 [3] | *“The evolutionary rationale for a Paleolithic diet and potential benefits were explained.”* | Over a 3-month study period, a Paleolithic diet improved glycemic control and several cardiovascular risk factors compared to a Diabetes diet in patients with type 2 diabetes. |
| Lindeberg et al 2007 [4] | *“Only subjects in the Palaeolithic group were educated in the concept of evolutionary health promotion and the potential benefits of a Palaeolithic diet.”* | A Palaeolithic diet may improve glucose tolerance independently of decreased waist circumference. |

References:

1. Jönsson T, Granfeldt Y, Lindeberg S *et al.* Subjective satiety and other experiences of a Paleolithic diet compared to a diabetes diet in patients with type 2 diabetes. *Nutr J* 2013;**12**:105.

2. Jönsson T, Granfeldt Y, Ahrén B *et al.* Beneficial effects of a Paleolithic diet on cardiovascular risk factors in type 2 diabetes: a randomized cross-over pilot study. *Cardiovasc Diabetol* 2009;**8**:35.

3. Jönsson T, Ahrén B, Pacini G *et al.* A Paleolithic diet confers higher insulin sensitivity, lower C-reactive protein and lower blood pressure than a cereal-based diet in domestic pigs. *Nutr Metab (Lond)* 2006;**3**:39.

4. Lindeberg S, Jönsson T, Granfeldt Y *et al.* A Palaeolithic diet improves glucose tolerance more than a Mediterranean-like diet in individuals with ischaemic heart disease. *Diabetologia* 2007;**50**:1795–807.
