# Supplementary material for: An evolutionary mismatch narrative to improve lifestyle medicine: a patient education hypothesis
Source: Evol Med Public Health. 2021 Feb 24;9(1):eoab010. doi: 10.1093/emph/eoab010 (PMC7962761; doi:10.1093/emph/eoab010)
Supplement: eoab010_Supplementary_Data [file eoab010_supplementary_data.zip › Supplemental Table 2_final.docx]

Supplemental Table 2. Evolutionary Narratives of Specific Hypothesized Mismatch Diseases

| **Pathology** | **Evolutionary Narrative** | **Treatment** |
| --- | --- | --- |
| Obesity | “There is an abundance of calories in our *modern environment*. These foods are extremely appetizing leading us to consume an excess of calories which contributes to obesity.” (Adapted from: [1]) | Limit processed food [2]; Restricting calories [3] |
| Diabetes | “Processed carbohydrates (like white bread) are quickly digested into sugar once you eat it. These types of food were not available in our *ancestors’ diets*. Eating too much of these foods for a long period of time can be problematic for your body.” (Adapted from: [4]) | Low glycemic load/index diets [5] |
| Depression | “*Modern society* is full of factors that impair our mental health, like perpetual upward comparison from media, fierce competition in a rapidly changing global economy, jobs performed in barren, confined spaces without clear, tangible results, and disrupted social support structures as our education and jobs pull us away from family.” (Adapted from: [6–8]) | Interpersonal therapy [9]; Therapeutic lifestyle change [10]; |
| Addiction | “Psychological mechanisms that were helpful in our *ancestral past* are now hijacked by supranatural signals like concentrated drugs, candy, and gambling that can lead to self-destructive behavior.” (Adapted from: [11–13]) | Replacement pharmacotherapy [14]; opioid receptor antagonism [15]; mindfulness-based interventions [16] |
| Insomnia | “Humans spend a lot of time inside in our *modern world*, away from sunlight. Further, computer and TV screens change our perception of daytime and nighttime. Together, these can harm your sleep pattern.” (Adapted from: [17]) | Limiting light pollution [18]; Limit blue light exposure [19] |
| Sedentarism | “We are inclined to conserve energy because food was hard to come by for our ancestors. The *modern environment* makes it easy to go places and do things without much physical effort. A lack of physical activity is a strong risk factor for a number of diseases.” (Adapted from: [20]) | Increasing physical activity [21] |
| Allergies | “Due to antibiotics, cleaning products, and waste infrastructure (like plumbing) *the modern environment* lacks many of the old microbial friends that help train our immune system. There are clear links between the loss of good bacteria and an increased risk of chronic inflammatory diseases, like asthma.” (Adapted from: [22,23]) | Limiting modifiable risk factors [24]; probiotics [25] |
| Note; italicized text reflects the evolutionary mismatch perspective. | | |

References:

1. Speakman JR. Evolutionary perspectives on the obesity epidemic: adaptive, maladaptive, and neutral viewpoints. *Annu Rev Nutr* 2013;**33**:289–317.

2. Hall KD, Ayuketah A, Brychta R *et al.* Clinical and Translational Report Ultra-Processed Diets Cause Excess Calorie Intake and Weight Gain: An Inpatient Randomized Controlled Trial of Ad Libitum Food Intake Cell Metabolism Clinical and Translational Report Ultra-Processed Diets Cause Excess Ca. *Cell Metab* 2019;**30**:1–11.

3. Zubrzycki A, Cierpka-Kmiec K, Kmiec Z *et al.* The role of low-calorie diets and intermittent fasting in the treatment of obesity and type-2 diabetes. *J Physiol Pharmacol* 2018;**69**, DOI: 10.26402/jpp.2018.5.02.

4. Eaton SB, Eaton SB. Physical Inactivity, Obesity, and Type 2 Diabetes: An Evolutionary Perspective. *Res Q Exerc Sport* 2017;**88**:1–8.

5. Augustin LSA, Kendall CWC, Jenkins DJA *et al.* Glycemic index, glycemic load and glycemic response: An International Scientific Consensus Summit from the International Carbohydrate Quality Consortium (ICQC). *Nutr Metab Cardiovasc Dis* 2015;**25**:795–815.

6. Hidaka BH. Depression as a disease of modernity: Explanations for increasing prevalence. *J Affect Disord* 2012;**140**:205–14.

7. Brenner SL, Jones JP, Rutanen-Whaley RH *et al.* Evolutionary Mismatch and Chronic Psychological Stress. *J Evol Med* 2015;**3**:1–11.

8. Li NP, van Vugt M, Colarelli SM. The Evolutionary Mismatch Hypothesis: Implications for Psychological Science. *Curr Dir Psychol Sci* 2018;**27**:38–44.

9. Feijo De Mello M, De Jesus Mari J, Bacaltchuk J *et al.* A systematic review of research findings on the efficacy of interpersonal therapy for depressive disorders. *Eur Arch Psychiatry Clin Neurosci* 2005;**255**:75–82.

10. Walsh R. Lifestyle and mental health. *Am Psychol* 2011;**66**:579–92.

11. Spinella M. Evolutionary mismatch, neural reward circuits, and pathological gambling. *Int J Neurosci* 2003;**113**:503–12.

12. Saah T. The evolutionary origins and significance of drug addiction. *Harm Reduct J* 2005;**2**:1–7.

13. Pani L. Is there an evolutionary mismatch between the normal physiology of the human dopaminergic system and current environmental conditions in industrialized countries? *Mol Psychiatry* 2000;**5**:467–75.

14. Nesse RM, Berridge KC. Psychoactive drug use in evolutionary perspective. *Science (80- )* 1997;**278**:63–6.

15. Sordo L, Barrio G, Bravo MJ *et al.* Mortality risk during and after opioid substitution treatment: systematic review and meta-analysis of cohort studies. *BMJ* 2017:j1550.

16. Chiesa A, Serretti A. Are Mindfulness-Based Interventions Effective for Substance Use Disorders? A Systematic Review of the Evidence. *Subst Use Misuse* 2014;**49**:492–512.

17. Nunn CL, Samson DR, Krystal AD. Shining evolutionary light on human sleep and sleep disorders. *Evol Med Public Heal* 2016;**2016**:227–43.

18. Chepesiuk R. Missing the dark: Health effects of light pollution. *Environ Health Perspect* 2009;**117**:20–7.

19. Tosini G, Ferguson I, Tsubota K. Effects of blue light on the circadian system and eye physiology. *Mol Vis* 2016;**22**:61–72.

20. Freese J, Klement RJ, Ruiz-Núñez B *et al.* The sedentary (r)evolution: Have we lost our metabolic flexibility? *F1000Research* 2017;**6**:1787.

21. Chaput JP, Lambert M, Mathieu ME *et al.* Physical activity vs. sedentary time: independent associations with adiposity in children. *Pediatr Obes* 2012;**7**:251–8.

22. Rook GAW. Review series on helminths, immune modulation and the hygiene hypothesis: The broader implications of the hygiene hypothesis. *Immunology* 2009;**126**:3–11.

23. Parker W, Perkins SE, Harker M *et al.* A prescription for clinical immunology: The pills are available and ready for testing. A review. *Curr Med Res Opin* 2012;**28**:1193–202.

24. Abreo A, Gebretsadik T, Stone CA *et al.* The impact of modifiable risk factor reduction on childhood asthma development. *Clin Transl Med* 2018;**7**, DOI: 10.1186/s40169-018-0195-4.

25. Zajac AE, Adams AS, Turner JH. A systematic review and meta-analysis of probiotics for the treatment of allergic rhinitis. *Int Forum Allergy Rhinol* 2015;**5**:524–32.
